# Lactate Metabolism and Satellite Cell Fate

**DOI:** 10.3389/fphys.2020.610983

**Published:** 2020-12-11

**Authors:** Minas Nalbandian, Zsolt Radak, Masaki Takeda

**Affiliations:** ^1^ Department of Clinical Application, Center for iPS Cell Research and Application (CiRA), Kyoto University, Kyoto, Japan; ^2^ Research Center of Molecular Exercise Science, University of Physical Education, Budapest, Hungary; ^3^ Graduate School of Sports and Health Science, Doshisha University, Kyoto, Japan

**Keywords:** lactate, muscle stem cell, metabolism, skeletal muscle, muscle regeneration

## Abstract

Lactate is one of the metabolic products of glycolysis. It is widely accepted as an important energy source for many cell types and more recently has been proposed to actively participate in cell-cell communication. Satellite cells (SCs), which are adult skeletal muscle stem cells, are the main players of the skeletal muscle regeneration process. Recent studies have proposed a metabolic switch to increase glycolysis in activated SCs. Moreover, lactate has been shown to affect SCs and myoblasts *in vivo* and *in vitro*. In this short review, we describe how metabolic variations relate with SC fate (quiescence, activation, proliferation, migration, differentiation, fusion, and self-renewal), as well as discuss possible relationships between lactate as a metabolite and as a signaling molecule affecting SC fate.

## Introduction

Satellite cells (SCs) are the main players responsible for the regeneration and maintenance of human adult skeletal muscle (Chargé and Rudnicki, 2004). During homeostasis, SCs are in a quiescent state with relatively low metabolic demands (Rodgers et al., 2014; Pala et al., 2018). Under stress, however, caused by injury, exercise, or other variables, SCs become activated to proliferate, differentiate, and fuse to regenerate the tissue. The transcriptional cascade that leads to these events has been deeply studied (Baghdadi and Tajbakhsh, 2018), but the mechanism that leads to these transcriptomic activations is not fully understood. Recent research has suggested that external factors such as metabolites or factors released by surrounding cells cause the activation of SCs (Tsuchiya et al., 2020; Zhang et al., 2020).

Interestingly, glycolysis has been related to many mechanisms that regulate epigenetic modifications in SCs (Ryall et al., 2015; Yucel et al., 2019). Lactate, which is produced by glycolysis, is easily transported between cells, making it a candidate mediator for these glycolysis effects. Furthermore, one recent study reported a new epigenetic modification process called lactylation, which is regulated by lactate (Zhang et al., 2019). However, little research has given attention to the possibility that lactate may regulate the epigenetic modifications that determine the SC state. In this short review, we summarize recent findings regarding SC metabolism and its effect on SC fate, with special consideration on lactate.

## Role of Satellite Cells in Skeletal Muscle

Healthy skeletal muscle is characterized by a high regenerative capacity (Chargé and Rudnicki, 2004). This regenerative capacity is attributed to the presence of SCs (Mauro, 1961). SCs are located beneath the basal lamina and close to the capillaries (Christov et al., 2007; Nederveen et al., 2016; Verma et al., 2018). Proper SC function is critical for not only the regeneration of adult skeletal muscle, but also its maintenance including muscle mass (Bentzinger et al., 2012).

During homeostasis, SCs exist in a quiescent state. Upon injury, SCs enter the cell cycle to become activated and undergo several rounds of proliferation, a process that happens very quickly from the 1st days after injury (Baghdadi et al., 2018). During proliferation, asymmetric and symmetric divisions happen. Symmetrically divided cells (mostly planar divisions) can give rise to two activated SCs or two SCs committed to myogenesis, while asymmetrically divided cells (mostly apical-basal division) will give rise to one activated SC and one quiescent SC, which contributes to the self-renewal of SCs (Cossu and Tajbakhsh, 2007; Kuang et al., 2007; Motohashi and Asakura, 2014). Activated SCs possess increased migratory capacity, which allows them to migrate to the injured sites (Baghdadi et al., 2018; Evano and Tajbakhsh, 2018). Once they reach there, the majority of activated SCs will start to differentiate into myoblasts and fuse to each other and to existing fibers to regenerate the injured site. A smaller population of active SCs will re-enter the quiescent state to maintain the SC pool (Rocheteau et al., 2012).

Satellite cells have been deeply studied, and the transcriptional program that regulates their fate has been established (Baghdadi and Tajbakhsh, 2018). Quiescent SCs are characterized by the expression of PAX7 (Seale et al., 2000), a paired-type homeobox transcription factor that is recognized as the major marker of adult SCs. After activation, PAX7 expression is decreased, and myogenic regulatory factors (MRFs) are expressed. MRFs are a family of basic helix-loop-helix (bHLH) transcription factors composed of MYF5, MYOD, MRF4, and MYOGENIN that promote the expressions of skeletal muscle-specific genes, determining which cells commit to myogenic differentiation (Pownall et al., 2002; Zammit, 2017; Asfour et al., 2018). When SCs become activated, MYF5 and MYOD expression is increased, while PAX7 expression decreases such that its protein level is undetectable in proliferating SCs. Later, cells that undergo differentiation will start expressing MYOG and MRF4 to exit the cell cycle and fully commit into myogenic differentiation (Baghdadi and Tajbakhsh, 2018).

Besides the transcription factors that determine SC fate, epigenetic changes in SCs have been shown to regulate myogenic differentiation (Liu et al., 2013; Wang et al., 2019; Shcherbina et al., 2020). Histones are proteins that provide structural support for packing DNA. Many histone modifications are known to be important for regulating transcriptional activity (Strahl and Allis, 2000). Consistently, when transitioning from the quiescent to activated state, SCs undergo several histone modifications associated with transcriptional changes that lead to myogenic differentiation (Liu et al., 2013). Furthermore, growing evidence has linked histone modifications with metabolism (Sabari et al., 2017).

## Metabolic Regulation of Satellite Cells During Regeneration

Different studies have shown that the metabolic profiles differ between quiescent, activated, and differentiated SCs, indicating that metabolic changes may affect SC fate (Ryall, 2013; Tang and Rando, 2014; Ryall et al., 2015; Pala et al., 2018; Yucel et al., 2019).

The majority of adult stem cells exist in a quiescent state and have low metabolic demand (Ito and Suda, 2014). SCs have no exception to this rule. It has been reported that after injury, SCs exit quiescence and increase their energy expenditure (Rodgers et al., 2014). Quiescent SCs have a low metabolism characterized by an increase in the expression of genes related to fatty acids oxidation (Fukada et al., 2007; Ryall et al., 2015), suggesting that lipids are the main source of energy for these cells. An RNA-sequencing (RNA-seq) analysis of freshly isolated mouse SCs (quiescent state) and cultured SCs (proliferating state) revealed that the expression of lipid metabolism-related genes is higher in the quiescent SCs than proliferating SCs (Fukada et al., 2007). Similarly, two recent studies (Ryall et al., 2015; Yucel et al., 2019) found that SCs when activated undergo a metabolic shift from lipids to glucose oxidation. When SCs exit the quiescent state, their energetic demands increase (Rocheteau et al., 2012; Rodgers et al., 2014; Dell’Orso et al., 2019; Purohit and Dhawan, 2019). Furthermore, it has been shown that quiescent SCs possess lower levels of mitochondrial activity than activated SCs (Rocheteau et al., 2012; Rodgers et al., 2014). Importantly, one single cell RNA-seq study of SCs in a skeletal muscle mouse injury model confirmed the increased expression of genes related to glycolysis and the tricarboxylic acid (TCA) cycle in activated SCs (Dell’Orso et al., 2019), suggesting that the higher energetic need of the activated SCs is fulfilled by aerobic and anaerobic pathways.

These metabolic changes have been proposed to play important roles in SCs fate. For instance, one study (Theret et al., 2017) suggested that AMP-activated protein kinase (AMPK), which is a master regulator of metabolic homeostasis (Hardie et al., 2012), regulates SC self-renewal by controlling metabolic homeostasis. In that study, AMPK knockout mouse SCs showed a high self-renewal rate and a switch of their metabolism to higher glycolysis, which impaired skeletal muscle regeneration. These findings provided evidence that metabolism and SC fate are connected. At the same time, AMPK has been shown to be regulated by nutritional strategies such as caloric restriction (Canto and Auwerx, 2011). In fact, caloric restriction has been shown to increase SC activity and to enhance skeletal muscle regeneration in mice (Cerletti et al., 2012), strengthening the hypothesis that metabolism regulates SCs fate.

**Figure 1:**
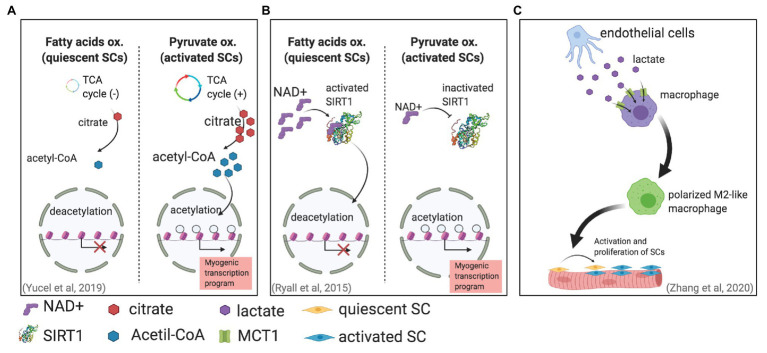
Different mechanisms in which metabolism can affect satellite cell (SC) fate. **(A)** SC activation is associated with an increase in pyruvate oxidation. This increase generates more citrate production, leading to more acetyl-CoA for histone acetylation that will enable gene transcription (Yucel et al., 2019). **(B)** Activated SCs increase pyruvate oxidation, reducing NAD+ and therefore SIRT1 activity. The result is higher histone acetylation and an activated myogenic transcriptional program. **(C)** Endothelial cells can secrete lactate that enters and induces the polarization of macrophages, supporting muscle regeneration by promoting SC proliferation and fusion (Zhang et al., 2020).

Metabolic changes associated with different SC states have been also correlated with chromatin modifications (Purohit and Dhawan, 2019). Histone acetylation, which is a chromatin modification associated with open chromatin and gene expressions, is regulated in part by the availability of acetyl-CoA (Wellen et al., 2009; Moussaieff et al., 2015). Furthermore, glucose is one of the main precursors for acetyl-CoA; during glycolysis, glucose produces pyruvate, which can be later converted into acetyl-CoA in mitochondria in a process mediated by pyruvate dehydrogenase (PDH). Thereafter, acetyl-CoA is converted to citrate, which can enter the TCA cycle and can be used to produce energy. Moreover, citrate is also transported out of mitochondria and diffuses to nuclei, where it can be used as a substrate to produce acetyl-CoA for histone acetylation. Interestingly, in proliferating SCs, an increase in glucose-derived citrate was found (Yucel et al., 2019). The same study found that this excess of citrate led to an increase in histone acetylation, further leading to a more accessible chromatin that ultimately modulated myogenic differentiation (Figure 1A).

Another mechanism has been proposed for the metabolically induced chromatin modifications in activated SCs (Ryall et al., 2015). Metabolic changes can regulate the NAD+-SIRT1 pathway; SIRT1 is a histone deacetylase that uses NAD+ as an enzymatic cofactor to accomplish histone deacetylation (Guarente, 2011; Jing and Lin, 2015). This mechanism, which links metabolic and stress changes with epigenetic modifications of the DNA, is of special relevance to maintaining genomic integrity (Bosch-Presegue and Vaquero, 2015). This mechanism has been suggested to be fundamental for regulating epigenetic processes in stem cells (Fang et al., 2019). Ryall et al. (2015) showed *in vitro* by using cultured mouse SCs and *in vivo* by using SIRT1 deacetylase domain ablation mouse models that the shift from fatty acid oxidation to glycolytic metabolism in activated SCs produces less intracellular NAD+ and consequently less SIRT1 activity, which ultimately leads to histone acetylation and the activation of muscle-specific gene transcription that promotes myogenesis (Figure 1B).

**Figure 2:**
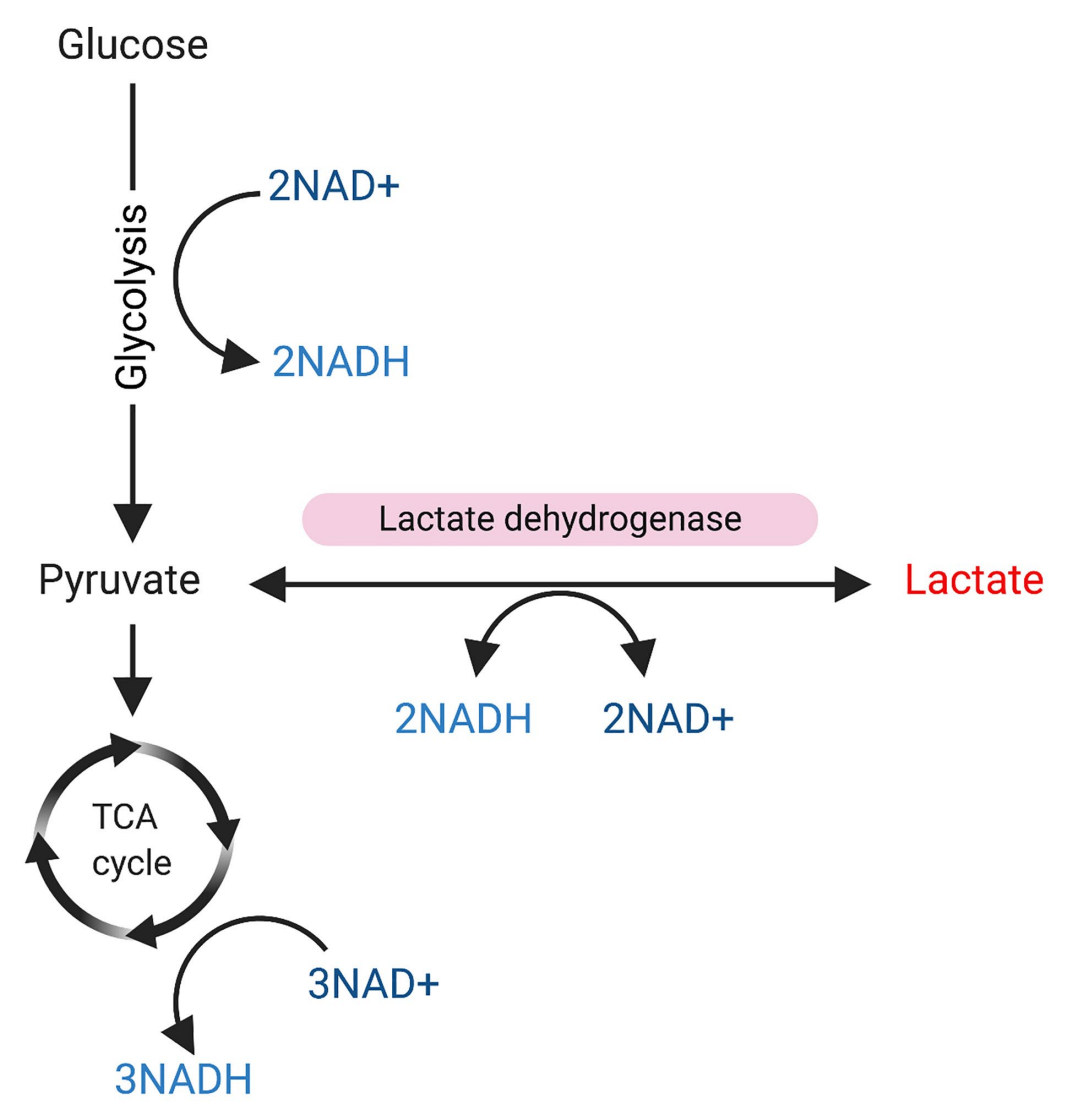
Glycolysis and sources of NAD/NADH+. Glucose is used as a substrate for glycolysis. During glycolysis 2 NAD+ will be converted to NADH, and pyruvate will be produced. Pyruvate can be used as substrate for the tricarboxylic acid (TCA) cycle, resulting in the conversion of 3NAD+ into NADH. On the other hand, pyruvate can be also converted into lactate in a reaction mediated by lactate dehydrogenase, which converts 2NADH to NAD+.

Given that lactate is a precursor of pyruvate and its role in reactions that involve NAD+/NADH conversion (see Figure 2), and also considering that the NAD+/NADH balance is critical for SIRT1 activation, it is reasonable to think that lactate may have some role in the metabolic control of SC fate with SIRT1 inactivation, favoring SCs activation. Indeed, some studies have suggested that lactate may affect SC commitment by inducing myogenic differentiation (Willkomm et al., 2014; Oishi et al., 2015; Ohno et al., 2019), but no study has reported a direct mechanism yet.

## Lactate as a Key Metabolite in the Control of Cell Signaling

Lactate is a metabolite produced from pyruvate by lactate dehydrogenase (LDH), with the LDH isoform A (LDHA) facilitating the pyruvate-to-lactate conversion in cells with high glycolytic rates, and the LDH isoform B (LDHB) facilitating the lactateto-pyruvate conversion in highly oxidative cells. When the cytoplasmic lactate concentration is elevated, lactate can be co-transported with one H+ ion outside the cell by facilitated diffusion *via* monocarboxylate transporters (MCTs; Halestrap and Wilson, 2012; Kitaoka et al., 2012; Halestrap, 2013; Perez-Escuredo et al., 2016). MCT1 and MCT4 are MCT isoforms expressed in skeletal muscle (Bonen, 2001). MCT1, which has a relatively low Km (3.5–10mM; Halestrap, 2012), is the predominant isoform in oxidative skeletal muscle fibers and considered responsible for lactate uptake (McCullagh et al., 1997; Juel and Halestrap, 1999; Pilegaard et al., 1999; Halestrap, 2012; Chatel et al., 2017). On the other hand, MCT4, which has a much higher Km (22–28mM; Halestrap, 2012), is the isoform predominantly expressed in glycolytic skeletal muscle fibers and considered responsible for lactate release (Dimmer et al., 2000; Fox et al., 2000; Bisetto et al., 2019). Extracellular lactate can travel through the blood stream to many cells, serving as an important energy source for several tissues and organs such as the brain (van Hall et al., 2009; Mosienko et al., 2015), liver, and skeletal muscle (Hui et al., 2017; Brooks, 2020). Given lactate’s ability to travel between cells, tissues, and organs, recently it was proposed to be a signaling molecule (Nalbandian and Takeda, 2016; Brooks, 2020).

The idea of lactate being a signaling molecule has been tested in several models. In differentiated C2C12 cells (mouse immortalized myoblasts), culture with L-lactate increased the expression of PGC1-alpha (peroxisome proliferator-activated receptor gamma coactivator 1-alpha), which is a transcription factor responsible for mitochondrial biogenesis (Nalbandian et al., 2019). Similarly, in differentiated L6 cells (immortalized rat myoblasts), culture with lactate increased the expression of PGC1-alpha as well as the expression of several genes related to metabolism like MCT1 (Hashimoto et al., 2007). Moreover, these results were confirmed *in vivo* by the intraperitoneal injection of lactate in mice (Kitaoka et al., 2016), where lactate could increase the skeletal muscle PGC1-alpha expression after 3 h. Additionally, in cancer cells (De Saedeleer et al., 2012; Colegio et al., 2014) and endothelial cells (Sonveaux et al., 2012), lactate induces the activation of hypoxia inducible factor-1 (HIF-1), which is a transcription factor recognized to have an important role in hypoxia and metabolism. Interestingly, HIF-1 has been shown to facilitate lactate transport in cancer cells by upregulating the expression of MCT4 (Ullah et al., 2006).

Together, the above studies indicate that on top of being an energy source, lactate can trigger several tissue-specific mechanisms that lead to the activation of diverse signaling pathways. Because of their location near blood vessels and capillaries (Christov et al., 2007; Verma et al., 2018), SCs may be exposed to high concentrations of lactate. Therefore, in the next section, we will discuss current evidence showing lactate affects SC fate.

## Possible Lactate Metabolism Effects on Satellite Cells

The effect of lactate on myogenesis has been studied *in vitro* and *in vivo*. In undifferentiated C2C12 myoblasts, culture with lactate and caffeine increased the number of proliferating cells and the number of nuclei per fiber in differentiated myotubes (Oishi et al., 2015). The same study showed that the lactate effect in myoblast differentiation was accompanied by an upregulation of mTOR and P70S6K phosphorylation, suggesting that lactate may work by activating anabolic signals in SCs. Similarly, a more recent study (Ohno et al., 2019) showed that differentiated C2C12 myoblasts cultured in differentiation medium containing lactate increased the size of myotube diameters and the number of nuclei per fiber. Moreover, the study showed that in mouse, oral administration of lactate stimulated muscle hypertrophy and increased the number of Pax7-positive cells, indicating the positive effect of lactate on skeletal muscle regeneration. In contrast, another study reported that lactate promoted the early stages of C2C12 differentiation in a reactive oxygen-dependent manner, but delayed the late stages, as indicated by the number of MHC-positive nuclei (Willkomm et al., 2014).

Another lactate-dependent mechanism that indirectly directs SC fate was reported recently. By using pfkfb3-deficient mice (pfkfb3 codes 6-phosphofructo-2-kinase/fructose-2,6-bisphosphatase 3 protein, which regulates glycolysis), Zhang et al. (2020) found that lactate secreted by endothelial cells was uptaken by and regulates the polarization of macrophages, which improved muscle reperfusion in hindlimb ischemia, and therefore promoted muscle revascularization and regeneration upon SC activation (Figure 1C).

Another mechanism by which lactate has been shown to affect macrophage polarity (which has not been related to muscle regeneration yet) is histone lactylation in which lactate mediates histone modifications (Zhang et al., 2019). That study correlated histone lactylation in macrophages during M1 polarization with the increased expression of genes that are involved in wound healing (i.e., genes that characterize M2 macrophages), suggesting a role in tissue homeostasis. It is unclear whether histone lactylation plays a role in skeletal muscle regeneration or if this histone modification is relevant for SC fate. Future studies should address these biological questions.

## Conclusion

Metabolism has been shown to be closely related with SC fate. A switch from fatty acids oxidation to glycolytic metabolism accompanies the transition from quiescent to activated SCs. This metabolic switch is also related to the redox balance produced by epigenetic changes *via* the NAD+-SIRT1 pathway and ultimately directs the fate of SCs to commit to myogenic differentiation. Lactate affects myoblast differentiation. Considering this, lactate’s close relationship with the redox balance and NAD+/NADH, and lactate’s mediation of histone modifications, the role of lactate in the myogenic differentiation of SCs should be considered in future investigations.

## Author Contributions

MN conceptualized the manuscript and wrote the manuscript. MT and ZR proofread the manuscript. All authors contributed to the article and approved the submitted version.

### Conflict of Interest

The authors declare that the research was conducted in the absence of any commercial or financial relationships that could be construed as a potential conflict of interest.
