# Corrigendum: Lactate Metabolism and Satellite Cell Fate

**DOI:** 10.3389/fphys.2021.817264

**Published:** 2022-02-15

**Authors:** Minas Nalbandian, Zsolt Radak, Masaki Takeda

**Affiliations:** ^1^Department of Clinical Application, Center for iPS Cell Research and Application (CiRA), Kyoto University, Kyoto, Japan; ^2^Research Center of Molecular Exercise Science, University of Physical Education, Budapest, Hungary; ^3^Graduate School of Sports and Health Science, Doshisha University, Kyoto, Japan

**Keywords:** lactate, muscle stem cell, metabolism, skeletal muscle, muscle regeneration

In the original article, there was an error. We state that LDHA isoform converts lactate to pyruvate and that LDHB converts pyruvate into lactate. The current evidence suggests that LDHA and LDHB both can favor the lactate to pyruvate conversion in any direction.

A correction has been made to “***LACTATE AS A KEY METABOLITE IN THE*** ***CONTROL OF CELL SIGNALING,” Paragraph 1***: “*Lactate is a metabolite produced from pyruvate by lactate dehydrogenase (LDH), with the LDH isoform A (LDHA) facilitating the pyruvate-to-lactate conversion in cells with high glycolytic rates, and the LDH isoform B (LDHB) facilitating the lactate-to-pyruvate conversion in highly oxidative cells. When the cytoplasmic lactate concentration is elevated, lactate can be co-transported with one H*+ *ion outside the cell by facilitated diffusion via monocarboxylate transporters (MCTs; Halestrap and Wilson*, *2012**; Kitaoka et al.*, *2012**; Halestrap*, *2013**; Perez-Escuredo et al.*, *2016**). MCT1 and MCT4 are MCT isoforms expressed in skeletal muscle (Bonen*, *2001**). MCT1, which has a relatively low Km (3.5–10 mM; Halestrap*, *2012**), is the predominant isoform in oxidative skeletal muscle fibers and considered responsible for lactate uptake (Mccullagh et al.*, *1997**; Juel and Halestrap*, *1999**; Pilegaard et al.*, *1999**; Halestrap*, *2012**; Chatel et al.*, *2017**). On the other hand, MCT4, which has a much higher Km (22–28 mM; Halestrap*, *2012**), is the isoform predominantly expressed in glycolytic skeletal muscle fibers and considered responsible for lactate release (Dimmer et al.*, *2000**; Fox et al.*, *2000**; Bisetto et al.*, *2019**). Extracellular lactate can travel through the blood stream to many cells, serving as an important energy source for several tissues and organs such as the brain (van Hall et al.*, *2009**; Mosienko et al.*, *2015**), liver, and skeletal muscle (Hui et al.*, *2017**; Brooks*, *2020**). Given lactate's ability to travel between cells, tissues, and organs, recently it was proposed to be a signaling molecule (Nalbandian and Takeda*, *2016**; Brooks*, *2020**).”*

The authors apologize for this error and state that this does not change the scientific conclusions of the article in any way. The original article has been updated.

## Publisher's Note

All claims expressed in this article are solely those of the authors and do not necessarily represent those of their affiliated organizations, or those of the publisher, the editors and the reviewers. Any product that may be evaluated in this article, or claim that may be made by its manufacturer, is not guaranteed or endorsed by the publisher.
